# A Case Series and Literature Review of the Association of COVID-19 Vaccination With Autoimmune Diseases: Causality or Chance?

**DOI:** 10.7759/cureus.28677

**Published:** 2022-09-01

**Authors:** Abdul-Wahab Al-Allaf, Maria Neethu, Yousr Al-Allaf

**Affiliations:** 1 Rheumatology, Hamad General Hospital, Doha, QAT; 2 Internal Medicine, Hamad General Hospital, Doha, QAT; 3 Medicine, Imperial College London School of Public Health, London, GBR

**Keywords:** covid-19 vaccine complication, covid vaccine-induced myocarditis, guillen barrie syndrome, jo1 syndrome, rheumatoid arthritis flare, systemic lupus erythromatosus, vasculitis, rheumatic disease & covid vaccines, autoimmune disease

## Abstract

The coronavirus disease 2019 (COVID-19) pandemic has been a challenging time for the whole world. Ever since the start of the pandemic, vaccine development has been underway and now there are several approved COVID-19 vaccines bringing hope for the end of the pandemic. However, there have been been a few individuals who have been affected in some other ways by the COVID-19 vaccinations. Here in this case series, we present 16 cases of autoimmune diseases with a strong temporal relation with the COVID-19 vaccine. We would like to emphasize that COVID-19 vaccines are essential to alter the course of the pandemic and save lives and the temporal relation is not by any means proof of causation. However, we must be vigilant for the occurrence of these conditions.

## Introduction

The coronavirus disease 2019 (COVID-19) pandemic has cost millions of human lives across the world. With recent advancements in vaccination and the approval of several vaccines across the globe, the mortality and morbidity rates have been effectively reduced. However, safety concerns regarding vaccines have been frequently encountered and there are several reports about new-onset autoimmune diseases following COVID vaccination. Molecular mimicry, the production of autoantibodies and the role of certain vaccine adjuvants seem to be some of the blamed causative factors of an autoimmune phenomenon [1].

However, it is not yet clear as to whether the occurrence of these autoimmune diseases is simply an incidental finding with a temporal relation, or if the relation is one of the causal associations. Here, we report a series of cases seen within our hospital with either new onset or reactivation of the autoimmune process after COVID-19 vaccination. We would like to point out that vaccinations are particularly recommended in patients with autoimmune conditions who are already immune-compromised, as they may experience more severe symptoms and harmful outcomes from the severe acute respiratory syndrome coronavirus 2 (SARS-Cov-2) infection course. Moreover, these patients are at a risk that their underlying autoimmune disease could be exacerbated following the infection with the SARS-CoV-2 infection [2].

## Materials and methods

Data was collected from our hospital between July 1, 2021, and December 31, 2021, of cases with either new autoimmune disease or exacerbation of the existing disease. Data was collected on initial outpatient or inpatient encounters and from our Cerner electronic health record system (Cerner Corporation, North Kansas City, Missouri, United States). The occurrence of exacerbation of the disease within six weeks after COVID-19 vaccination was considered as a temporal relation. All our cases received either Pfizer-BioNTech COVID-19 Vaccine or Moderna COVID-19 (mRNA-1273) vaccine except for one patient who had the Astra-Zeneca vaccine.

The study got approval from ABHATH, Hamad Medical Corporation, Qatar (ID MRC-04-22-415) and was conducted in Hamad General Hospital, Doha, Qatar.

## Results

Inflammatory arthritis and COVID-19 vaccination

Case 1

A 65-year-old male with seronegative rheumatoid arthritis, previously treated with rituximab and maintained on sulfasalazine, was in complete clinical remission for more than two years. He developed joint pain and swelling within a few days after the first dose of the COVID-19 vaccine and continued to progress to a severe flare of his arthritis within a week after receiving the second dose of the Pfizer BioNTech COVID-19 vaccine. He was clinically having multiple painful, swollen, and tender joints with significant stiffness with a high erythrocyte sedimentation rate (ESR) and C-reactive protein (CRP). The intramuscular steroid has been given and the treatment was escalated by increasing the dose of the sulfasalazine with a good response. After good control of his disease, he managed to receive the third vaccine dose with no problem. After eight months of continuous treatment escalation with sulfasalazine to the maximum dose of 3g daily and monitoring, his disease was brought under control.

Case 2

A 55-year-old male with palindromic rheumatism with positive rheumatoid factor (RF) and Anti-cyclic citrullinated peptide (anti-CCP) was well-controlled on hydroxychloroquine (HCQ) for more than a year with no flare-ups. He then developed a flare of his arthritis a week after his first dose of COVID-19 vaccination (Pfizer BioNTech), but this time with multiple joint pain, swelling, and tenderness. Musculoskeletal ultrasound (MSK US) confirmed widespread active synovitis and he was accordingly transferred from a well-controlled simple palindromic rheumatism to a full-blown seropositive rheumatoid arthritis. He was treated with steroids and non-steroidal anti-inflammatory drugs (NSAIDs) with added methotrexate. After optimizing his methotrexate dose, he was brought under good control, with minimal disease activity.

Case 3

A 31-year-old female was previously fit and developed acute onset inflammatory oligoarthritis three weeks after the first dose of COVID-19 vaccination (Pfizer BioNTech). She was started on NSAIDs and prednisone 5mg from a private clinic with no response. On evaluation, she had active arthritis of the right knee, ankle, wrist, and left elbow, with laboratory test results showing a CRP of 15, negative RF, anti-citrullinated peptide antibody (ACPA), and antinuclear antibodies (ANA). She was given a higher reducing dose of steroids and NSAIDs and successfully responded to it. It was stopped after a few weeks with no recurrence on reviewing her two months after stopping it.

Case 4

A 71-year-old male with diabetes, hypertension, and chronic kidney disease developed multiple joint pain and swelling including small joints of the hands, wrists, shoulders, knees, and feet within two weeks of the COVID-19 vaccination. His initial CRP was 95 and he was given a short course of steroids. However, he continued to have active arthritis and his CRP increased to 102; RF, ACPA, and ANA were negative. He was eventually started on HCQ and steroids were tapered off and stopped over two months. We reviewed him four months after that with no recurrence of his inflammatory arthritis and with normalization of his inflammatory markers. We continued his HCQ for a total of six months only.

Case 5

A 37-year-old female, who is a known case of psoriasis under dermatology follow-ups, developed multiple joint pains and swellings (proximal interphalangeal (PIP) joints, wrists, ankles, and both Achilles's tendons) within 10 days of her second dose of COVID-19 vaccination. CRP was 86 and ESR 40 with negative RF, ACPA, and ANA. She was given a short course of steroids and NSAIDs and responded to it.

Case 6

A 31-year-old male, who was previously fit and well, developed right ankle pain and swelling on the first day after his first COVID-19 vaccination. The pain was severe to the point that he was unable to weight-bear and required regular NSAIDs. MSK US confirmed active synovitis/tenosynovitis and joint effusion. He improved with the NSAID course; however, he had recurrent symptoms after the second dose, for which he again needed a course of NSAIDs and gradually improved. Although his condition was settled, he noticed that he develops ankle pain during activities. An MRI revealed mild tendonitis. He is now much better and off medications.

COVID-19 vaccination and systemic lupus erythematosus (SLE)

Case 7

A 51-year-old female patient of SLE was in remission for the past seven years and completely off medications. She developed a flare of SLE with the same symptoms of her initial presentation including inflammatory polyarthritis, recurrence of her multiple mouth ulcers, hair loss, photosensitivity, and leukopenia after her second dose of COVID-19 vaccination (Pfizer BioNTech). Laboratory test results showed WBC of 3.7, normocytic anaemia, high ESR of 45, and normal CRP, C3, and C4. She was given a short course of steroids by the general practitioner with complete resolution of her symptoms. However, her symptoms recurred after stopping steroids and she was urgently seen at our rheumatology outpatient clinic. On examination, she had inflammatory arthritis (joints pain, swelling, and tenderness), hair loss, and mouth ulcers. She was re-started back on steroids with added HCQ and needed to continue on a low-dose steroid (5 mg daily) and HCQ to see improvement clinically and lab-wise.

Case 8

A 26-year-old female who was diagnosed of SLE/mixed connective tissue disease was in remission on HCQ 200 mg and azathioprine (AZA) 100 mg since August 2020. She also has had a previous deep vein thrombosis (DVT) with factor V Leiden variant and negative antiphospholipid syndrome (APS) screening and was on rivaroxaban. She received the first dose of the Pfizer BioNTech COVID-19 vaccine in March 2021. She developed lower back pain with vaginal and gum bleeding 10 days after the vaccination and attended the Emergency Department. Laboratory test results showed thrombocytopenia (with initial platelets of 64,000, which further dropped to 49,000), low C3 and C4, and normal Anti-double-stranded (Ds) DNA. She was given IV methylprednisone 60 mg for three days followed by oral prednisone 30 mg daily with a good response (her platelet count recovered to 240,000). Her second vaccination dose was postponed, which enabled us to taper off steroids aiming for a better vaccine response. She received the second vaccine dose and her platelets remained stable, staying above the level of 155,000.

COVID-19 vaccination and vasculitis

Case 9

**Figure 1:**
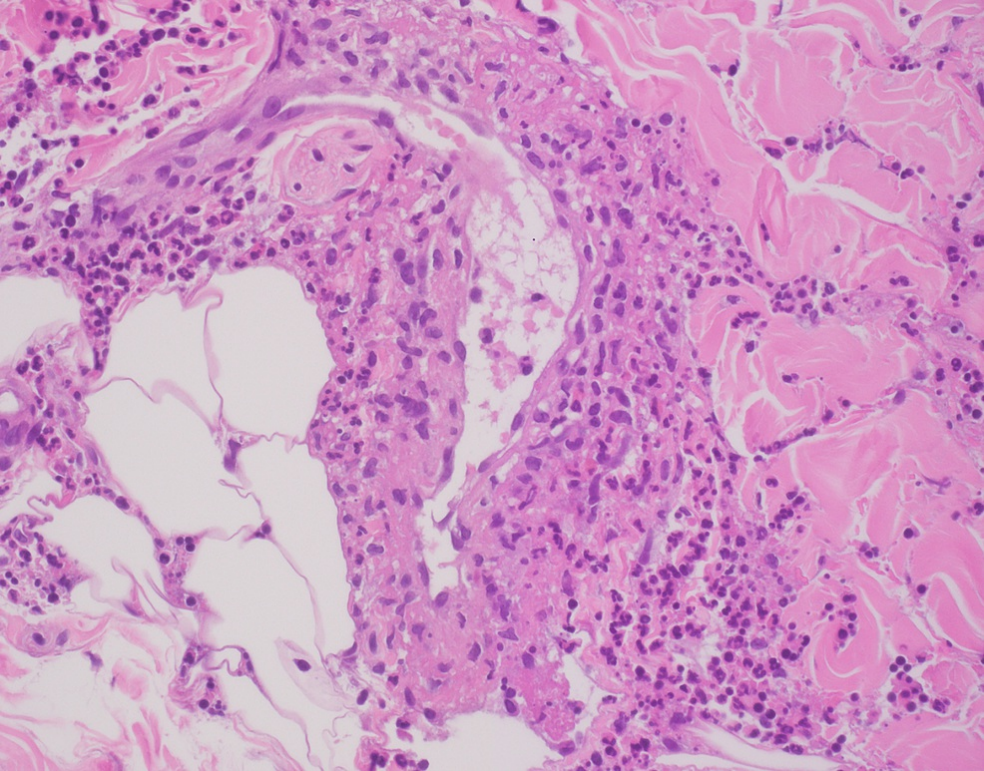
Histological findings of Case 9: Dermal small vessel vasculitis with neutrophilic infiltrate is seen, which is characteristic of leukocytoclastic vasculitis

A 27-year-old female who was previously fit and well, presented with a skin rash affecting both lower limbs four weeks after her second dose of COVID-19 vaccination (Pfizer BioNTech). Examination showed non-blanching maculopapular red lesions up to her knee with right ankle synovitis. Her clinical picture was suggestive of leukocytoclastic vasculitis with right ankle arthritis. Laboratory test results showed normal total cell counts, high platelets at 500,000, haemoglobin of 11.8g/dL and high inflammatory markers with a CRP of 74 and ESR of 42. ANA and ANCA were negative, and C3 and C4 were normal. She was started on prednisone 20 mg a day, with a tapering dose and topical steroids with good clinical improvement. She had a biopsy of her skin lesions, which showed neutrophilic infiltrate with occasional eosinophils within the wall of dilated blood vessels with interstitial fragmented neutrophilic nuclei (leucocytoclasis). Overall histological findings were of dermal small vessel vasculitis with neutrophilic infiltrate and leucocytoclastic vasculitis (Figure 1).

Case 10

**Figure 2:**
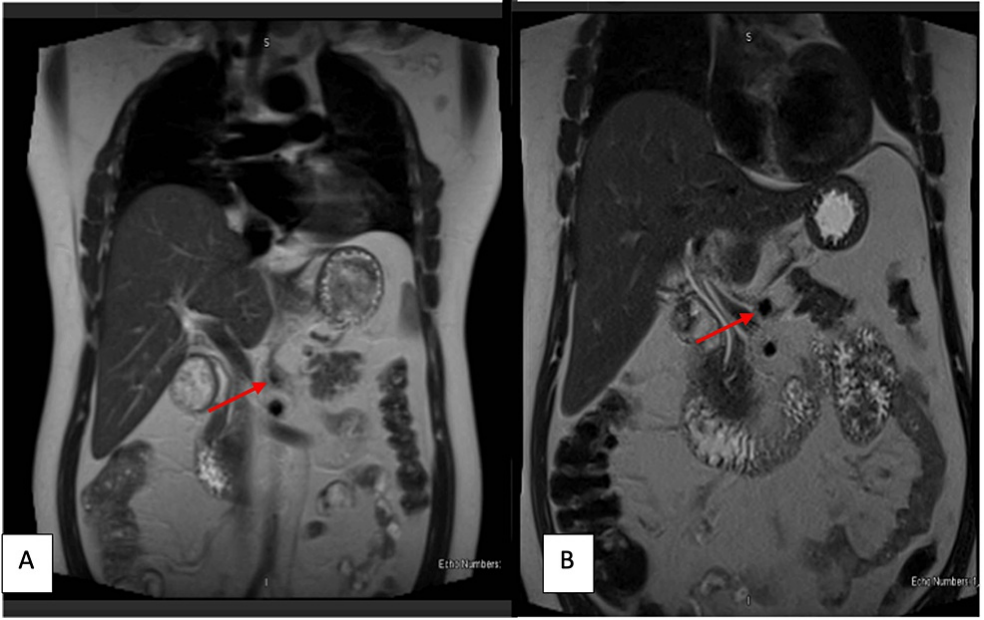
(A) Initial MRA Aorta of Case 10 showing coeliac artery with narrowing and irregular thickened wall due to surrounding inflammation (arrowhead); (B) Post-treatment MRA of Case 10 showing regression of the mural and perivascular thickening involving the coeliac trunk and its branches with the restoration of its lumen (arrowhead). MRA: magnetic resonance angiography

A 46-year-old male who was a chronic smoker and hypertensive presented to the Emergency Department with a history of severe epigastric pain for the past five days not responding to analgesics. It started a day after the second dose of Pfizer BioNTech COVID-19 vaccination [3]. His laboratory test results showed normal complete blood count, renal and liver function, and levels of ferritin, amylase, and lipase with a raised ESR of 11 and CRP of 20. Urgent CT abdomen with contrast revealed diverticulosis with no evidence of inflammation and isolated dense fat stranding surrounding the celiac artery trunk and its major branches, likely representing focal vasculitis. He was reviewed by the rheumatology department for workup. Further autoimmune workup including ANA, ENA panel, ANCA, RF, and anti-CCP was negative. QuantiFERON (QIAGEN, Hilden, Germany), Hepatitis B and C, urine, stool, and blood cultures were all negative. His upper gastrointestinal endoscopy and gastric biopsy were normal with negative *Helicobacter pylori*. Magnetic resonance angiography (MRA) of the abdomen was subsequently done and showed narrowing and irregularity of the celiac trunk (Figure 2a), splenic, hepatic, and left gastric arteries, with features of underlying vasculitis or IgG4-related disease and pancreatic lesion with recommendation for dedicated MRI of the pancreas. This was done later and ruled out any pancreatic lesions. Immunoglobulins including IgG and IgG4 were normal. The patient was started on pulse methylprednisone followed by high-dose oral steroids and AZA. He improved clinically and was followed in regular outpatient follow-ups with a good resolution of his symptoms. He developed one episode of acute red eye afterward and was seen by an ophthalmologist with the impression of acute iritis, which was treated with a course of topical eye drops on top of his above medications. Repeat imaging of the MRA Abdomen was done six months later, which showed that there is a regression of the mural and perivascular thickening involving the celiac trunk and its branches with the restoration of its lumen, which suggests improvement of the underlying inflammatory process (Figures 2b).

Case 11

A 63-year-old female with diabetes and hypertension presented with a new-onset left-sided temporal headache, which started soon after her first dose of COVID-19 vaccination. She also had complaints of jaw claudication, proximal muscular pain, stiffness, and inflammatory arthralgia. She was seen by an ophthalmologist for blurred vision and was told she had diabetic retinopathy. On examination, she had scalp tenderness on the left side, laboratory test results showed a high ESR of 150 mm/hr (her previous baseline was 25 mm/hr). She was started on oral prednisone 60 mg daily with a good response to symptoms and ESR dropped to 33 mm/hr. A good temporal artery biopsy of 2 cm revealed no evidence of giant cell arteritis and she was treated as a case of polymyalgia rheumatica (PMR) with possible temporal arteritis. Given her normal temporal artery biopsy, she was treated with 40 mg of prednisolone, which was reduced quickly over a month to 15 mg (the dose for PMR) and then received a more gradual reduction after that. We managed to reduce the dose to 5 mg in about three months with complete resolution of her symptoms. About a month later, she developed a flare-up of her typical polymyalgia symptoms, which responded very well to 120 mg intramuscular (IM) depomedrone. Two months later, she developed another flare of her typical PMR symptoms in association with jaw claudication and scalp tenderness, which also responded well to a booster dose of 120 mg IM depomedrone.

Case 12

A 46-year-old lady who is known to have hyperlipidemia on rosuvastatin 10 mg for the last three years got vaccinated with the COVID-19 vaccine because she had to travel. Within a few hours of receiving her first Moderna vaccine, she developed a severe diffuse allergic skin reaction with on and off hives, which could involve every part of her body including around the eyes, ears, palms, and soles. Some lesions looked like erythema multiformis, others were typical of hives with linear and occasional nodular raised and confluent lesions. Investigations revealed hypochromic microcytic anemia with Hb 10.8, ESR 8, CRP 5.6, with negative RF and ANA, and normal LFT, TFT, renal profile, Vitamin D, and normal creatine kinase (CK). She was seen in the allergy department and treated with prednisolone 40 mg daily for a week with excellent response. However, as soon as she stopped the steroid, she started to have a typical inflammatory pain all over the body including muscles, joints, and even the spine with no associated joint swelling. She has significant nocturnal and early morning stiffness and stiffness after rest, which improved to some extent with physical activities and as the day went on. She had difficulty sleeping because of the severe pain and stiffness and she was crying and even frightened to go to sleep because of that. She was given another reducing dose of steroid for about a month with some help. But soon after stopping the steroid, she seized again with the generalized aches and pain and stiffness. We saw her two months after her initial vaccination with the main complaint of generalized inflammatory aches and pain and stiffness with no associated joint swelling. All her joints had the full range of movement and there was nothing to suggest temporal arteritis. We gave her 120 mg IM depomedrone, which led to 60-70% improvement of her aches and pain. However, even after six months following her vaccine, she continued to have the on and off skin lesions with hives. Her symptoms seemed to be similar to that of PMA, despite her normal inflammatory markers and her age. We decided to give her another IM depomedrone if needed. A deferral certificate was initiated on her initial presentation and she was advised to avoid further doses of Moderna.

Case 13

**Figure 3:**
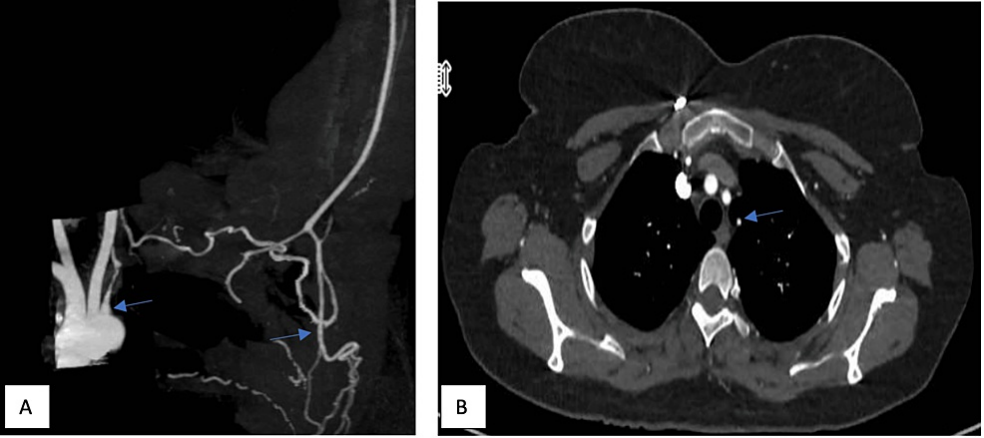
Maximum intensity projection (MIP) coronal image (A) and axial thin cuts (B) of CT angiography in Case 13 showing complete obliteration of the left subclavian artery after the origin of the left vertebral artery (arrow) with severe attenuation of the proximal left subclavian artery and vertebral artery; (A) Reconstitution of the left upper limb arterial supply from the cervicothoracic collaterals from the axillary artery onwards

A 43-year-old female, with a history of bronchial asthma and left vertebral artery occlusion, was diagnosed 25 years ago in her home country (with no medical reports available) and she was not on any chronic medications. She presented with claudication pain in her left upper limb. Her symptoms started two weeks after the second dose of the Pfizer BioNTech COVID-19 vaccination. She was evaluated by the Vascular team and CT angiography was done, which showed complete obliteration of the left subclavian artery and irregular circumferential narrowing of the right subclavian artery with subtle wall enhancement (Figure 3). Findings likely represent arteriopathy, likely Takayasu arteritis. Hence, she was referred to the Rheumatology team. On examination, she had an absent left radial pulse and a weak right radial pulse. Laboratry test results showed normal ESR and a CRP of 10mg/L. She was started on a reducing dose of prednisone 30 mg daily along with AZA 50 mg once daily resulting in good improvement of her symptoms. However, on reducing the steroid from 20 to 15 mg, she developed the recurrence of her symptoms with raised inflammatory markers, both ESR and CRP. Prednisolone was put back to 20 mg and the AZA dose was increased to 100 mg daily. 

COVID-19 vaccination and Jo1 syndrome

Case 14

A healthy 47-year-old lady received two doses of the COVID-19 vaccine. The first vaccine was Pfizer and the second one was Astra Zeneca, which she received in August 2021. Four weeks after her second vaccine, she presented to a private clinic with a significant problem with skin rash over the dorsum of her metacarpophalangeal (MCP) and PIP joints and an inflammatory joint pain, which was initially thought initially to be skin psoriasis with psoriatic arthritis. She was started on methotrexate in a private clinic. At the same time, she complained of cough and shortness of breath and was seen by a respiratory physician and a chest high resolution CT (HRCT) scan was arranged. She also complained of dryness in her skin and eyes with swelling around her eyes with some discoloration that was unusual for her. She also complained of significant inflammatory myalgia, but with no significant muscular weakness. The rash over her MCP and PIP joints is typical for Gottron's patch. Interestingly, her blood tests revealed strongly positive anti-Ro and positive J01 antibody, raised CRP and platelets, high IgE at 302 kunits/L (normal up to 114) with negative tests for allergens and RF, and normal CK. Her HRCT revealed minimal infiltrates in the right middle lung lobe and bilateral minimal bronchiectasis and subsegmental atelectasis in the middle lung lobe and lingula of the left upper lobe as well with bilateral minimal apical fibrosis, which indicated interstitial lung disease. She continued with the methotrexate. Initially, she was given 120 IM depomedrone injections on two occasions four weeks apart with excellent response to her skin dryness and rash, arthritis and myalgia. In her last visit, five months after her initial presentation, she continued to be well but complained of mild pain and stiffness in her hands, which occured more during the night and early morning associated with some tingling and numbness in the median nerve distribution on both sides. We arranged for her to have a nerve conduction study and she was given wrist splints. Her methotrexate was increased to 20 mg once a week with the folic acid 5 mg two days after the methotrexate dose and started on vitamin D 50,000 international units once a week. Accordingly, we diagnosed her as having post-COVID-19 vaccine J01 syndrome (arthritis, inflammatory myalgia, Gottran' patch, positive J01, strongly positive anti-Ro antibody, raised CRP and platelet, with excellent response to steroid).

COVID-19 vaccination and Guillain-Barré syndrome

Case 15

An 80-year-old female with well-controlled diabetes and hypertension was followed up in Rheumatology for osteoporosis. She developed severe weakness in both lower limbs one week after the Pfizer BioNTech COVID-19 vaccination, to the extent that she was unable to stand from a sitting position. She was assessed by the Neurology team and nerve conduction studies confirmed Guillain-Barré syndrome, which was treated with IV methylprednisolone and IVIG. She needed rehabilitation care for about three months with slow progress. No CSF analysis has been done. She continued to have some weaknesses with no complete resolution. 

COVID-19 vaccination and myocarditis

Case 16

**Figure 4:**
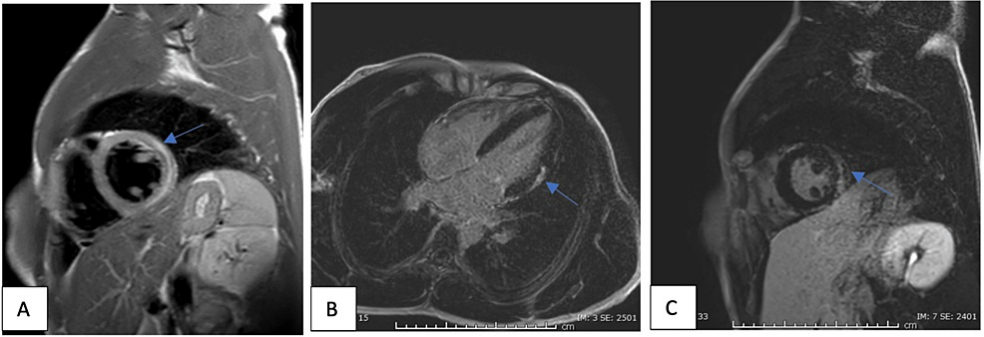
(A) T2 weighted selective short-axis image of Case 16 showing evidence of hyperintense signal at the basal to the mid-level lateral wall extending to the anterolateral walls impressive of myocardial edema; (B) Post-gadovist late enhancement four-chamber view images of Case 16 showing subepicardial to mid-wall enhancement basal to mid-level at the lateral walls extending to the anterolateral wall, which is in keeping with recent myocarditis; (C) Post-gadovist late enhancement short-axis view images of Case 16 showing subepicardial to mid-wall enhancement basal to mid-level at the lateral walls extending to the anterolateral wall, which is in keeping with recent myocarditis

**Figure 5:**
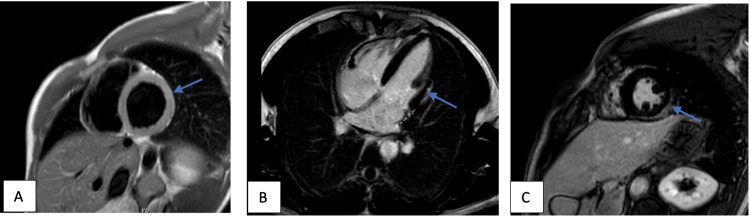
(A) T2 weighted selective short-axis images of Case 16 showing resolution of previously seen myocardial edema; (B) Post-gadovist late enhancement four-chamber view images of Case 16 showing mid-lateral and inferior wall subepicardial fibrosis; (C) Post-gadovist late enhancement short-axis view images of Case 16 showing mid-lateral and inferior wall subepicardial fibrosis

A 29-year-old male who was previously healthy presented two weeks after his COVID-19 vaccination (Moderna) with palpitations. Laboratory test results showed high inflammatory markers and troponin and he was strongly triple-positive for antiphospholipid antibodies without thrombosis. Subsequently, an MRI heart was done, which showed evidence of myocarditis (Figure 4). He was started on colchicine, NSAIDs, and beta-blockers. A follow-up MRI showed resolution of his myocarditis and the patient improved clinically as well (Figure 5). 

## Discussion

In our case series, we reported four cases of new-onset inflammatory arthritis, two cases of a flare-up of underlying arthritis, three cases of vasculitis, two cases of a flare-up of SLE, and two cases of PMR with possible temporal arteritis in one of them, all after COVID-19 vaccination. We also reported one case of Guillain-Barré syndrome, one case of Jo-1 syndrome, and one case of myocarditis with strongly triple-positive antiphospholipid antibodies without thrombosis.

There has been emerging evidence regarding COVID-19 vaccination and the potential trigger of autoimmune conditions. In this report, we described the cases that we encountered at our hospital, which were found to have a temporal correlation between vaccine administration and the development of symptoms of autoimmune diseases. A review of the literature showed various case reports and studies with conflicting results. As with our cases, Ishay et al. reported eight similar cases including the temporal arteritis-like disease [4]. Terracina et al. described a case of a patient with rheumatoid arthritis who was in remission for a long time and subsequently flared up soon after the COVID-19 vaccination [5]. Patil and Patil described a case report of new-onset SLE soon after COVID-19 vaccination [6]. Renal limited ANCA vasculitis after vaccination has been also reported following the COVID-19 vaccination [7]. Another case of ANCA-associated vasculitis with rhabdomyolysis and pauci-immune crescentic glomerulonephritis has been reported post-vaccination [8]. There have also been several reports about leucocytoclastic vasculitis post COVID-19 vaccination [9-11]. Concerning autoimmune neurological manifestations after COVID-19 vaccination, there have been several reports in the literature of Guillain-Barré syndrome, as in our case [12]. 

A prospective analysis of 77 patients with rheumatoid arthritis by Bixio et al. confirmed a low flare rate after COVID vaccination [13]. The international vaccination against COVID in systemic lupus (VACOLUP) cross-sectional study, which included 696 participants, concluded that the risk of a flare of SLE after COVID-19 vaccination was minimal [14].

Chin et al. have proposed that the potential mechanism by which the COVID-19 vaccine triggers autoimmunity includes molecular mimicry, leading to the production of particular autoantibodies and a possible role of certain vaccine adjuvants [15]. Previously, it has been well proposed that Influenza, hepatitis B, and human papillomavirus vaccines trigger autoimmunity through molecular mimicry [16]. 

It is too early to comment on whether these post-COVID-19 vaccine autoimmune diseases are short-lived or not, as most of our cases seem to need long-term therapy such as the cases of vasculitis, temporal arteritis with the PMR, and also the Jo1 syndrome patients. Some of the reactivated inflammatory arthritis patients required increases in their immunosuppression therapy. 

Despite the good temporal association, we cannot presume for sure that the vaccine is the causative agent for these occurrences. To confirm that vaccination has a causative role in the development of these autoimmune diseases, there is a need for well-designed prospective post-marketing studies. This association also needs to be confirmed at a molecular level. Till then, we must be more vigilant for such associations and have a high index of suspicion in considering the possibility of such diseases after the COVID-19 vaccine, and keep reporting such possible adverse events.

The benefits of the COVID-19 vaccine outweigh the possible associated risks on both the individual and community levels. However, with more emerging cases of autoimmune-related diseases following COVID-19 vaccination, publications of the COVID-19 vaccines' post-marketing surveillance results are to be done as a matter of urgency. We also urge the vaccine developers and drug companies to publish the full results for any reported side effects at the earliest. National and international post-marketing registries can help a lot in answering some of these questions and it may give light on who is at a higher risk of such autoimmune disease associations. 

## Conclusions

Possible complications of COVID-19 vaccines have become of special interest to both healthcare workers and the public. Our report showed a temporal relation between the COVID-19 vaccination and the occurrences or flare of autoimmune conditions. However, this should not be deemed to signify causality. Moreover, considering that immunosuppressed patients are at higher risk of having a more severe COVID-19 infection, such patients and the general public should be vaccinated. However, we advise our colleagues to remain vigilant for such association, so that any potential increased risk of flare or new-onset autoimmune phenomenon is promptly diagnosed, evaluated, and treated. We also need more post-marketing studies to check who is at a higher risk of such possible vaccine-related side effects and how we can avoid that.
